# Efficient ultrafast laser writing with elliptical polarization

**DOI:** 10.1038/s41377-023-01098-2

**Published:** 2023-03-15

**Authors:** Yuhao Lei, Gholamreza Shayeganrad, Huijun Wang, Masaaki Sakakura, Yanhao Yu, Lei Wang, Dmitrii Kliukin, Linards Skuja, Yuri Svirko, Peter G. Kazansky

**Affiliations:** 1 Optoelectronics Research Centre, University of Southampton, Southampton, SO17 1BJ UK; 2 Institute of Solid State Physics, University of Latvia, 8 Kengaraga Strasse, Riga, LV-1063 Latvia; 3 Institute of Photonics, Department of Physics and Mathematics, University of Eastern Finland, FI-80101 Joensuu, Finland

**Keywords:** Optical data storage, Laser material processing

## Abstract

Photosensitivity in nature is commonly associated with stronger light absorption. It is also believed that artificial optical anisotropy to be the strongest when created by light with linear polarization. Contrary to intuition, ultrafast laser direct writing with elliptical polarization in silica glass, while nonlinear absorption is about 2.5 times weaker, results in form birefringence about twice that of linearly polarized light. Moreover, a larger concentration of anisotropic nanopores created by elliptically polarized light pulses is observed. The phenomenon is interpreted in terms of enhanced interaction of circularly polarized light with a network of randomly oriented bonds and hole polarons in silica glass, as well as efficient tunneling ionization produced by circular polarization. Applications to multiplexed optical data storage and birefringence patterning in silica glass are demonstrated.

## Abstract (web summary)

Efficient creation of anisotropic nanopores and associated birefringence in fused silica by elliptically polarized femtosecond laser pulses is observed. Applications in multiplexed optical data storage and optical elements are demonstrated.

## Graphical abstract

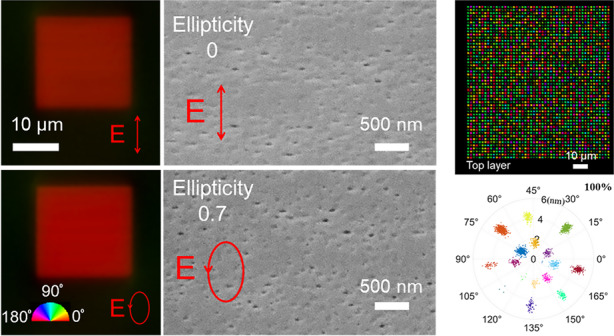

## Introduction

The photoexcitation, and especially photoionization, is one of the most important manifestations of the light–matter interaction in nature, ranging from photosynthesis in plants and vision in biology to applications in photography and laser processing of materials^1,2^. It is commonly accepted that the less light absorbed by the photosensitive material, the weaker the permanent change. Here, in experiments on ultrafast laser processing of materials, we have found that this is not always the case.

Progress in high-power ultrashort pulse lasers, e.g., chirped pulse amplification (CPA)^3^, has opened new frontiers in physics and the technology of light–matter interactions. The interaction of intense ultrashort light pulses in particular with transparent materials has attracted considerable interest due to the observation of new phenomena^4–7^ and a wide range of applications^8^ in laser surgery^9^, three-dimensional integrated optics^10–13^, microfluidics^14,15^, direct printing of optical elements and optical data storage^16^. The interaction is initiated by multiphoton absorption in the focus of the light beam, followed by free-electron creation and energy transfer to the lattice without collateral damage of the material. One of the unsolved puzzles in this process is the observation of self-assembled nanolamella structures (referred to as nanogratings or type II modification) aligned perpendicular to laser beam polarization of a size as small as 20 nm in the volume of some transparent materials and, in particular, silica glass^17–19^. These nanostructures are volumetric in contrast to the polarization-dependent periodic patterns induced on the surface of metals^20^, semiconductors^21^, and dielectrics^22^ under the irradiation with a linearly polarized light beam. Despite several attempts to explain the peculiar process of self-organization in bulk glass, the mechanism of formation of these volumetric nanostructures remains debatable.

Regardless the particular mechanism of subwavelength structures formation they are capable to make the medium anisotropic depending on their shape and/or mutual arrangement^23,24^. Optical anisotropy is defined as dependence of the optical properties on the direction of propagation and polarization of light in the medium. In non-gyrotropic crystals, the linear birefringence is a measure of optical anisotropy. Induced (artificial) optical anisotropy in media that are by nature optically isotropic arises upon exposure to external stimuli, which could be dc or ac electric field (the Kerr effect), magnetic field (the Cotton-Mouton and Faraday effects), or stress (the photoelastic effect). The induced optical anisotropy can be transient or persistent due to a permanent change or alignment in the molecular structure^25–27^.

Similar to other birefringence patterning methods such as alignment of liquid crystals^27^ and lithography^28^, the birefringence in silica glass associated with the nanostructures has found applications for polarization shaping, geometric phase optics and microfabrication^29,30^. More recently, subwavelength nanostructures have been explored for polarization-multiplexed optical data storage, where encoding information with three spatial coordinates is enhanced by adding two birefringence parameters, i.e., the slow axis orientation (4th dimension) and strength of retardance (5th dimension)^31,32^. Compared to other multiplexed data storage techniques that, e.g., employ fluorescence of sliver clusters embedded in glass^33^, plasmonic properties of metallic nanoparticles^34,35^ and laser writing in optically active polymers^36^ or transparent plastics^37^, the 5D optical data storage based on direct writing in silica glass offers the high data capacity and virtually unlimited lifetime^32^. One of the challenges for the 5D optical data storage is increasing the writing speed, which is limited by the high driving voltage of electro-optic modulators used to control polarization. Moreover, low transmission, especially in the ultraviolet and visible domains, can limit the application of birefringence patterning. Recently, ultra-high transmission birefringent modification consisting of randomly distributed oblate nanopores(type X) was observed in silica glass, which has been used for high efficiency (>99%) phase and polarization shaping^38^ and more recently for data storage^39^.

Most of the research has been done with linearly polarized laser pulses, and only a few experiments have used circular and elliptical polarizations. Ultrafast laser writing with circular polarization revealed the formation of randomly distributed spherical nanoparticles on the surface of CaF_2_^40^, ZnO and ZnSe crystals^41^. There has been demonstrated the formation of one-dimensional periodic surface structures oriented perpendicular to the long axis of the polarization ellipse^40,41^ and an increase in the structure period on a silicon surface with increasing ellipticity^42^. The capillaries were fabricated in silica glass by selective etching assisted by femtosecond laser direct writing and controlled by the ellipticity of the laser beam^43^.

The polarization dependence of photoionization was investigated experimentally in fused silica, and it was shown that at relatively low intensities (<15 TW/cm^2^), the photoionization rate is ~3–4 times greater for linearly polarized radiation than for circular polarization^44^. For higher intensities (>35 TW/cm^2^) the photoionization rate for circular polarization becomes greater due to the contribution of tunneling ionization. A stronger index change was observed in the silica glass irradiated with a circularly polarized light at an intensity of 45 TW/cm^2^ ^45^. Nevertheless, in other transparent materials irradiated in the conditions of predominant multiphoton ionization, no increase in the index modification was reported with increasing ellipticity of light.

Although one may expect that by optical means the strongest nanopore-mediated birefringence can be created by a linearly polarized light beam, here we report that in silica glass, elliptical polarization is more efficient even despite lower absorption. The phenomenon is interpreted in terms of enhanced interaction of circularly polarized light with randomly oriented bonds and hole polarons in silica glass network as well as a high probability of tunneling ionization for low excitation energy defects by circular polarization. Birefringence patterning with elliptical polarization results in an increased throughput in the fabrication of polarization shaping and geometric phase optical elements. Multilayer 5D optical data storage with elliptical polarization is demonstrated with tens of kB/s writing speed and nearly 100% readout accuracy.

## Results and discussion

**Fig. 1:**
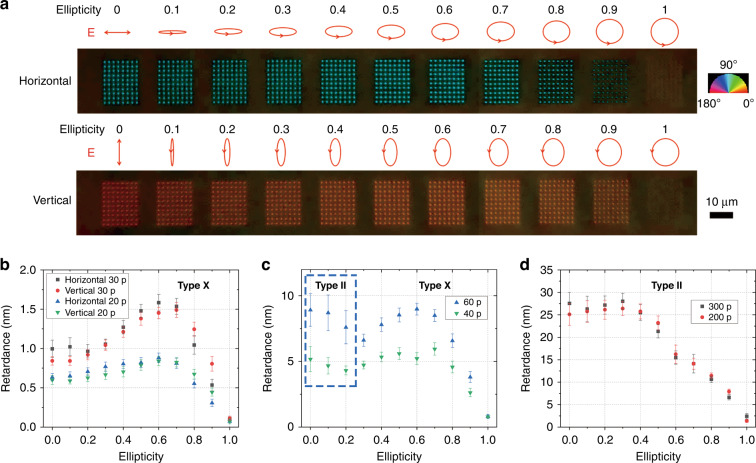
Femtosecond laser direct writing of birefringent modification by different ellipticities of the beam. **a** The birefringence image of voxels imprinted by 30 pulses. Pseudo colors (inset) indicate the local orientation of the slow axis. **b** Retardance of type X modification as a function of ellipticity for 20 or 30 pulses with the energy of 200 nJ. **c** and **d** Retardance for birefringent voxels versus ellipticity with pulse energy of 220 nJ at different pulse numbers. Processing conditions: 1 MHz repetition rate, 300 fs pulse duration, 1030 nm wavelength, 0.3 NA lens

Birefringent voxels with a high transmittance and a slow axis oriented perpendicular to the laser polarization direction were imprinted by laser pulses with the energy of 200 nJ (intensity 9.8 TW/cm^2^), a repetition rate of 1 MHz and a pulse duration of 300 fs. Light pulses with two orthogonal polarizations and light ellipticities varying from 0 (linear polarization) to 1 (circular polarization) were used for writing 80 birefringent voxels with 2 μm lateral separation (Fig. 1a). The background between voxels was removed by an algorithm for the precise retardance measurement^39^. The retardance of birefringent voxels produced with 30 linearly polarized pulses was about 1 nm, and no birefringence was observed with circular polarization (Fig. 1b). With ellipticity up to 0.2, the retardance was close to the value for a linearly polarized beam. However, starting from a light ellipticity of 0.3, the retardance began to increase^46^, reaching a maximum value of 1.5 nm at an ellipticity of about 0.6, followed by a decrease at higher ellipticity (Fig. 1b). Similar trend was observed with 20 pulses, but the rate of increase from linear to elliptical polarization was lower than for the case of 30 pulses.

Birefringent voxels were also imprinted at a higher energy of 220 nJ (10.8 TW/cm^2^) when the nanopore modification (type X) was transformed into the nanograting modification (type II). When the number of pulses exceeded 40, the type II modification was observed for an ellipticity of less than 0.4, however, interestingly, type X voxels were produced with a more elliptical polarization (Fig. 1c). Usually, anisotropic nanopores transform into nanogratings at higher pulse energy, a larger number of pulses, or a longer pulse duration^38^, but here we have demonstrated that such a transformation can also be realized by decreasing the ellipticity of the beam. At more than 200 pulses, only birefringent voxels with nanograting type modification were observed, with almost no change in retardance at an ellipticity of less than 0.4 and a subsequent decrease at higher ellipticity (Fig. 1d). An increase in retardance with increasing ellipticity of light is a property of the type X modification, suggesting a peculiar mechanism for the formation of anisotropic nanopores, which is different from the self-organized mechanism of nanogratings formation.

**Fig. 2:**
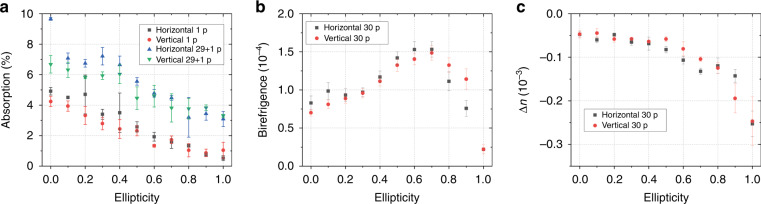
Optical properties of voxels as a function of light ellipticity. **a** Measured single laser pulse absorption versus ellipticity. **b** Birefringence and **c** index change of voxels imprinted by 30 pulses with different ellipticities. Processing conditions: 1 MHz repetition rate, 300 fs pulse duration, 1030 nm wavelength, 200 nJ pulse energy, 0.3 NA lens

To understand the physical mechanism of nanopore formation, nonlinear absorption measurements were carried out. The absorption of a single pulse with a duration of 300 fs and an energy of 200 nJ for linear and circular polarization was 5% and 1%, respectively (Fig. 2a). This measurement is consistent with the fact that multiphoton absorption (eight photons of 1.2 eV for silica glass) with an ionization probability at an intensity of 9.8 TW/cm^2^ higher for linear polarization than for circular polarization^44^. Since dozens of laser pulses are required to record one birefringent voxel, we also measured the absorption for the 30th laser pulse as a function of ellipticity (Fig. 2a). The first 29 pulses were focused inside the sample, and a few seconds later, the 30th pulse was triggered in single pulse mode to measure its absorption. The absorption of the last pulse in the sequence was stronger than that of the first pulse, which can be explained by the accumulation of defects^47^ and also decreased with increasing ellipticity of the beam. The birefringence can be estimated from the measured retardance and the length of the structure (Δ*n*_*b*_ = *Ret*./*l*). Contrary to intuition, we demonstrate that light pulses with ellipticity as high as 0.6 can produce voxels with a birefringence of 1.5 × 10^−4^, which is 1.9 times greater than the value of 0.8 × 10^−4^ for linearly polarized pulses (Fig. 2b), despite 2.5 times lower absorption of light with elliptical polarization (5% for the linear polarization and 2% for the ellipticity of 0.6). Non-zero birefringence with circular polarization is explained by the slightly elongated shape of voxels along the scanning direction. We estimated a negative index change as high as −2.5 × 10^−4^ (−0.5 × 10^−4^) for circular (linear) polarization (Fig. 2c).

**Fig. 3:**
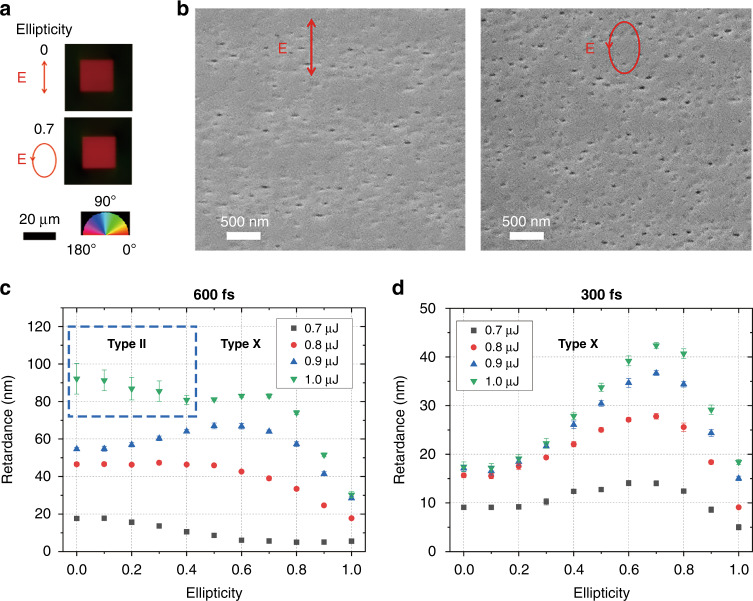
Birefringence laser patterns written with different ellipticities and energies. **a** Birefringence and **b** scanning electron microscope (SEM) image of the region imprinted by linearly (top) and elliptically (bottom) polarized laser pulses with a duration of 600 fs and energy of 0.9 μJ. **c** and **d** Retardance of birefringent modification versus ellipticity with pulse duration of 600 fs and 300 fs. Writing conditions: 10 mm/s scanning speed, 200 kHz repetition rate, 1030 nm wavelength, 0.16 NA lens

Laser-induced ultra-low loss birefringence in silica glass can be used for the fabrication of vector beam converters and geometric phase optical elements^38,48^. However, the printing of type X elements requires a relatively long manufacturing time. To avoid the transition to type II modification, the maximum retardance of a single layer type X for optical elements fabrication is about 55 nm by using linearly polarized 600 fs pulses with an energy of 0.9 μJ and translating speed of 10 mm/s. Inspired by voxel recording, we investigated the effect of elliptical polarization on the imprinting of birefringent elements. Birefringent square structures of 20 × 20 µm in size were written by raster scanning (Fig. 3a) with a line spacing of 1 µm using different ellipticities, durations and energies of laser pulses. Similar to voxel recording, the retardance of birefringent square written by elliptically polarized pulses is increased to 70 nm, compared to 55 nm written by linear polarization. Different from voxel writing, non-zero retardance was observed in square writing with circularly polarized pulses, which can be attributed to stress-induced birefringence due to volume change surrounding laser written tracks. However, its slow axis orientation is not dependent on polarization direction of writing laser beam.

The SEM images of the high transmission birefringent modifications after polishing and etching were taken to reveal the effect of light ellipticity on nanopore formation (Fig. 3b). It should be noted that the nanopores sizes estimated from SEM images are larger than their real size due to etching step. Randomly distributed anisotropic nanopores with a width of about 20–30 nm were observed in the region irradiated with linearly polarized pulses. In comparison, nanopores with a size of 30–40 nm and a larger density were created by pulses with elliptical polarization, leading to a larger retardance of birefringent modification. Without KOH etching, the actual size of nanopores written by linear (elliptical) polarization could be just 15 (25) nm. The nanopores were flattened in the direction perpendicular to the major axis of the polarization ellipse of the laser beam, which is the cause of the birefringence. Although the larger concentration of anisotropic nanopores was observed in birefringent square imprinted by elliptically polarized pulses, its optical transmission (98.4% at 550 nm) is similar to those imprinted by linearly polarized pulses (98.6% at 550 nm).

The transition from modification of the nanograting type II to the nanopore type X was observed with a change in the ellipticity of the beam at a pulse duration of 600 fs and energy of 1 μJ (intensity 8.6 TW/cm^2^). The maximum retardance of 83 nm for the modification of type X at a light ellipticity of 0.6 is almost 1.5 times higher than the retardance produced by a linearly polarized light (Fig. 3c), which can be used to reduce the manufacturing time of optical elements. No retardance increase with polarization ellipticity was observed for pulse energy less than 700 nJ due to low light intensity of 6.0 TW/cm^2^. At a pulse duration of 300 fs, the retardance increases with increasing ellipticity, and the maximum value of 42 nm appears at an ellipticity of 0.7, which is about 2.5 times the value obtained at linear polarization (Fig. 3d). The ratio of retardance for structures written with elliptical and linear polarization increases with increasing pulse energy.

The mechanism of the observed phenomenon can be explained as follows. It has already been demonstrated that oblate nanopores are responsible for a high transmittance birefringence in the silica glass irradiated with linearly polarized pulses^38^. The nanopores are flattened as the result of the anisotropy of near-field enhancement with linear polarization. The question then arises why these anisotropic nanopores are more efficiently created by pulses of elliptically polarized light despite a lower ionization probability? If we consider an elliptically polarized pulse as a combination of linear and circular polarized components, then it is reasonable to assume that the linear component is responsible for the flattening of nanopores due to the anisotropy of the near-field, and the circular component is responsible for a more efficient formation of nanopores. The latter is confirmed by a decrease of the refractive index indicating that the cavitation and associated reduction of density^49,50^ dominate the material modification produced by a circularly polarized component.

In the case of photoionization by a circular polarization, electrons are always accelerated away from the ion acquiring kinetic energy, which is twice the ponderomotive energy (*U*_*p*_)^51,52^. By contrast, with linearly polarized laser pulses, free electrons are left with low kinetic energy, less than photon energy, because they experience alternative acceleration and deceleration by the laser field during each optical cycle of the pulse. To our conditions (intensity 11 TW/cm^2^ and wavelength 1030 nm), we estimate the average energy of free electrons of 0.34 eV for the linear polarization while it increases to 2.15 eV for the circular polarization^52^. However, it turns out that due to collisions, the electron heating rate in solids does not depend on the light ellipticity.

Another possibility may be related to the polarization dependence of nonlinear excitation efficiency of randomly oriented dipolar absorbing species—point defects or localized states introduced by glassy disorder^53^. Having lower excitation energies than the band gap of silica, they are expected to serve as initial sites for nonlinear ionization. The probability of n-photon excitation of a dipole oriented at an angle α to the electric field is proportional to cos^2n^(α)^54^. Defects can also be excited more efficiently by tunneling, which is in line with our experimental conditions given low excitation potentials (<2 eV) and high light intensities (~10 TW/cm^2^). Interestingly, the probability of tunneling ionization may be higher for circular polarization than for linear polarization, e. g. for the hydrogen atom, the probability ratio between two polarizations ($\sqrt {\pi /3F}$, *F* is electric field strength in atomic units) is about 8 for the light electric field 0.015 of the atomic field^55^. Moreover, circularly polarized light with its rotating electric field direction can thus access a larger part of the statistically oriented localized states compared to linearly polarized light. In this way, the circular component of elliptically polarized light may serve to maximize the number of photoionized sites, while the linear component provides the anisotropy, necessary to create birefringence.

In particular, non-bridging oxygen hole centres (NBOHC, ≡ Si–O∙), oxygen dangling bonds^53,56^, formed during nonlinear photoionization of silica glass have a low energy absorption band at 2 eV. A rotating electric field of circular polarization efficiently reduces the trapping barrier, which results in tunneling excitation of NBOHC producing a hole in the valence band. Other common defects with low excitation energy are self-trapped holes (STHs, hole-based polaron) with absorption bands ranging from 0.6–2.6 eV^57,58^. Generally, small imperfections of the SiO_2_ network due to the glassy disorder (“glass localized states”) are necessary for stable STHs to form: in the regular lattice of α-quartz holes remain mobile even at 4 K^59^.

STHs as well as NBOHCs are produced and accumulated in a series of intense light pulses with a wavelength of 1030 nm. Subsequent pulses can excite STHs by tunneling, which results in activation of holes. We assume that the probability of excitation of STHs with randomly oriented Si–O bonds increases for pulses with circular polarization. Activation of hole-based polarons can possibly lead to clustering, which minimizes the energy of interaction between positive holes and the lattice and promotes the formation of nanopores.

The birefringence of the anisotropic modification based on nanopores can be explained by two factors: one is the form birefringence associated with the boundary conditions at the interface of the nanostructure, and the other is the birefringence caused by stress in the volume surrounding the nanostructure due to the change in density. The strength of both types of birefringence will increase with increasing concentration, size, and shape anisotropy of nanoporous structures and contribute to the observed increase in birefringence with elliptical polarization. However, the contribution of stress-induced birefringence to the measured birefringence is only 5–10% for type II modifications based on nanogratings^24^. As the stress in type X modifications is less than in type II structures, the stress-induced birefringence contribution to the measured birefringence of the type X modification must be less than that of their type II counterparts. Therefore, the observed increase in birefringence with elliptical polarization should be mainly due to the form birefringence and not to stress-induced birefringence, as confirmed by annealing experiments (Supplementary Section 1).

**Fig. 4:**
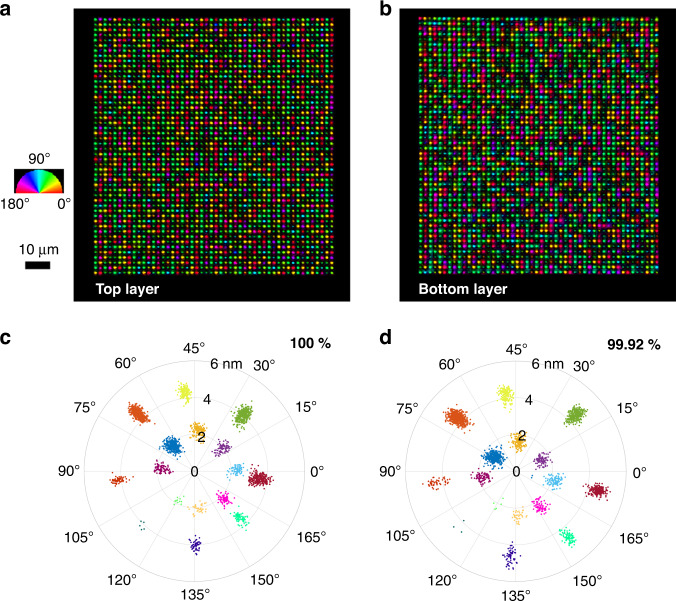
50 layers of 5D optical data storage by elliptical polarization writing. **a**, **b** The birefringence image of data voxels from the top and bottom layer. 4 bits per voxel were realized by 2 retardance and 8 azimuth levels. **c**, **d** Polar diagram of the measured retardance and azimuth of all voxels in (**a**) and (**b**). Writing conditions: 1 MHz repetition rate, 300 fs pulse duration, 45 mm/s translating speed, 215 nJ pulse energy, 20 pulse number, 0.3 NA lens

A copy of a digital document was written in 50 layers from bottom to top as a demonstration of elliptical polarization writing by two Pockels cells (Supplementary Section 2). Two levels of retardance were obtained with two different light ellipticity values (0.6 and 0.8) with an energy of 215 nJ and a pulse number of 20 (Fig. 4a, b), which is reduced from 30 for data recording with linearly polarized pulses. Two retardance levels, as well as eight azimuths of the slow axis, were demonstrated (Supplementary Section 2), meaning that 4 bits of information (2^4^ = 2^1^ × 2^3^) can be encoded into one voxel of 5D optical data storage. The data readout accuracy of the 1^st^ layer and the 50^th^ layer was 100% (Fig. 4c) and 99.92% (Fig. 4d), respectively. The lateral voxel separation and layer separation was 2 μm and 17.5 μm, respectively, leading to the data capacity of 350 GB in a 5-inch disc and the data writing speed of 11 kB/s. By reducing the separation of voxels and layers down to 0.5 μm and 5 μm, respectively, one can achieve the data capacity per 5-inch disk as high as ~20 TB. The data readout speed, which is currently limited by the commercial liquid crystal based birefringence imaging system, can be increased up to MB/s by using Pockels cells and computer-controlled motorized imaging system.

Modulating the retardance with polarization ellipticity is also more practically attractive, as about 1.5 times higher data writing speed can be achieved by reducing the number of pulses from 30 to 20. In addition, thermal damage from high repetition rate pulses can be mitigated by elliptical polarization writing due to low absorption, which could increase the data recording rate by a factor of 5 (Supplementary Section 3). Thus, the write speed for elliptical polarization can be improved by a total factor of 10, up to 250 kB/s at a repetition rate of 5 MHz, and with ten channels of parallel recording, more than 2.5 MB/s can be realized.

## Conclusion

In conclusion, we have demonstrated an efficient creation of anisotropic nanopores and related birefringence in silica glass using elliptically polarized laser pulses. Contrary to common belief, the maximum induced birefringence occurs at an ellipticity of 0.6 and not at linear polarization. This is a consequence of the balance between the concentration of nanopores with a maximum at circular polarization and their shaping due to the anisotropy of the near-field enhancement produced by the linear polarization component. It is suggested that enhanced interaction of a circularly polarized beam with randomly oriented bonds and activation of self-trapped holes in silica glass, as well as more efficient tunneling ionization of defects with low excitation energy by circular polarization are responsible for this phenomenon. In addition, the voltage required to control the elliptical polarization is significantly reduced, which allows the use of electro-optical modulators with higher modulation frequency for birefringence patterning. Writing with elliptically polarized laser pulses makes it possible to reduce the pulse energy and increase the recording speed in 5D optical data storage, as well as reduce the manufacturing time of geometric phase optical elements.

## Materials and methods

The experiments were carried out with a mode-locked regeneratively amplified femtosecond laser system (PHAROS, Light Conversion Ltd.), operating at a wavelength of 1030 nm with a repetition rate varying from 200 kHz to 1 MHz and a pulse duration tuning in the range of 190–300 fs. Laser pulses were focused with a 0.30 or 0.16 NA (numerical aperture) aspheric lens 170 μm beneath the surface of a synthetic silica glass sample, which was placed on a three-axial air-bearing translation stage (Aerotech Ltd). The ellipticity of the laser beam without changing the azimuth was controlled by a single Pockels cell. A universal compensator, consisting of a linear polarizer, a quarter-wave plate, and a pair of Pockels cells with an angle of 45° between their main axes, was used to create an arbitrary direction of polarization and ellipticity of the laser beam, which were measured using a polarimeter (PAX1000, Thorlabs).

The retardance and slow axis orientation of the birefringent voxels were quantified with a birefringence measurement system (Abrio, Cri. Inc) operating at 546 nm wavelength attached to an Olympus BX51 optical microscope. The phase change was measured with a wavefront sensor (SID4-HR, Phasics) mounted on the same microscope. After polishing and etching with a KOH solution (1 mol/L) for 24 h, the laser-modified region was imaged by a scanning electron microscope (SEM, Zeiss Leo 1450).

## Supplementary information

Supplementary Information for Efficient ultrafast laser writing with elliptical polarization

## Data Availability

All data are available in the main text or the supplementary materials from the corresponding author upon reasonable request.
