# Supplementary material for: Efficient ultrafast laser writing with elliptical polarization
Source: Light Sci Appl. 2023 Mar 15;12:74. doi: 10.1038/s41377-023-01098-2 (PMC10015004; doi:10.1038/s41377-023-01098-2)
Supplement: Supplementary file 1 — Supplementary Information for Efficient ultrafast laser writing with elliptical polarization [file 41377_2023_1098_MOESM1_ESM.docx]

Supplementary Information for

Efficient ultrafast laser writing with elliptical polarization

Yuhao Lei,^1,🖂^ Gholamreza Shayeganrad,^1^ Huijun Wang,^1^ Masaaki Sakakura,^1^ Yanhao Yu,^1^ Lei Wang,^1^ Dmitrii Kliukin,^1^ Linards Skuja,^2^ Yuri Svirko,^3^ and Peter G. Kazansky^1, 🖂^

*^1^Optoelectronics Research Centre, University of Southampton, Southampton, SO17 1BJ, United Kingdom*

*^2^Institute of Solid State Physics, University of Latvia, 8 Kengaraga str., LV1063, Riga, Latvia*

*^3^Institute of Photonics, Department of Physics and Mathematics, University of Eastern Finland, FI-80101 Joensuu, Finland.*

^🖂^Correspondence: Yuhao Lei ([yuhao.lei@soton.ac.uk](mailto:yuhao.lei@soton.ac.uk)) or Peter G. Kazansky ([pgk@soton.ac.uk](mailto:pgk@soton.ac.uk))

1. **Annealing experiment**

The measured birefringence of type II and type X modifications can be attributed to both form birefringence of nanostructures and stress-induced birefringence in the volume surrounding nanostructure due to density change. The strength of both types of birefringence will increase with the increase of the concentration, size and the anisotropy of the shape of nanopores. Since the concentration of nanopores is larger when the elliptically polarized pulses were used to imprint type X modifications, we observed the increase of birefringence with elliptical polarization. It has been demonstrated that the contribution of stress-induced birefringence to the measured birefringence is as low as 5-10% for nanograting based type II modifications [[1](#_ENREF_1)]. Since the stress in type X modification is smaller than their type II counterparts, the contribution of stress-induced birefringence should be even smaller.

The annealing experiments were performed to confirm our interpretation. Birefringent voxels with a high transmittance (type X) were imprinted by 30 or 40 laser pulses with different polarization ellipticities from 0 (linear polarization) to 1 (circular polarization), the energy of 210 nJ (intensity 10.3 TW/cm^2^), a repetition rate of 1 MHz and a pulse duration of 300 fs. Hereafter, the silica glass sample with birefringent voxels was heated at rate of 1000 ℃ per hour, kept at 800 ℃ for two hours and cooled to room temperature at rate of 100 ℃ per hour. With the same heating and cooling rates, the sample was annealed at 1050 ℃ for 30 minutes as well. It is clear that after annealing at 800 ℃ for 120 minutes, the retardance of voxels decreases very little and the tendency of retardance versus ellipticity is same as the voxels before annealing. For higher annealing temperature of 1050 ℃ and annealing time of 30 minutes, around 50% percent stress-induced birefringence should be eliminated [[1](#_ENREF_1)]. After annealing at 1050 ℃, the retardance drops around 10% compared to the voxels before annealing and the dependence of the retardance versus ellipticity of polarization is the same as before. Therefore, we can conclude the birefringence increase with elliptical polarization is mainly attributed to the form birefringence instead of stress-induced birefringence.


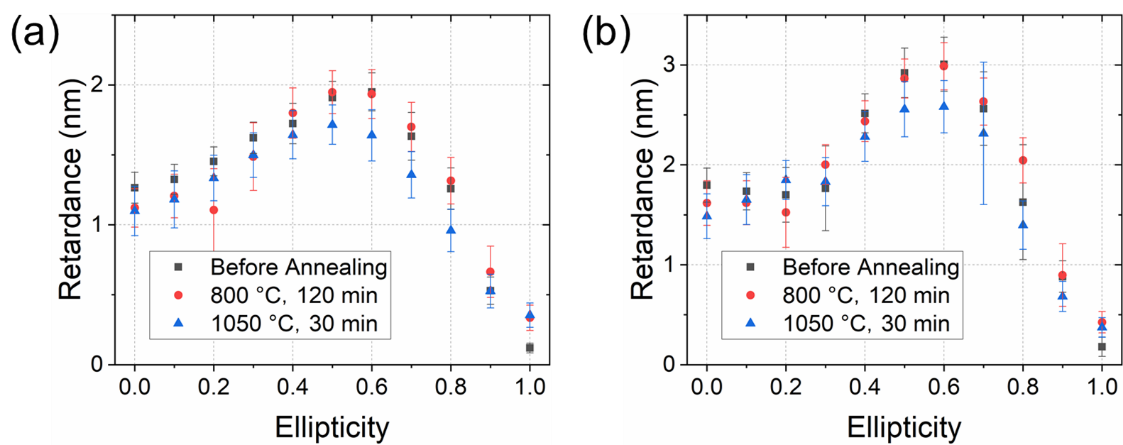


Fig. S1. Measured retardance of voxels versus ellipticity of polarization before and after annealing. The number of writing pulses are 30 (a) and 40 (b), respectively. The annealing was carried out at 800 ℃ for 120 minutes and 1050 ℃ for 30 minutes. Processing conditions: 1 MHz repetition rate, 300 fs pulse duration, 1030 nm wavelength, 210 nJ pulse energy, 0.3 NA lens.

1. **Polarization control**

The ellipticity of polarization without changing the azimuth was controlled by a single Pockels cell. A universal compensator, consisting of a linear polarizer, a quarter-wave plate, and a pair of Pockels cells with an angle of 45° between their main axes, was used to create an arbitrary direction of polarization and ellipticity of the laser beam, which were measured using a polarimeter (PAX1000, Thorlabs).

A polarization multiplexed data writing in the bulk of silica glass was used for a demonstration of the application of the observed phenomenon. The polarization ellipticity [Fig. S2a] and polarization azimuth of the laser beam [Fig. S2b] were measured with a polarimeter and controlled by changing the phase in the range from -0.15π to 0.12π with the first Pockels cell (PC1) and from -0.2π to 0.2π with the second (PC2), respectively. The beam was focused with a 0.3 NA lens and birefringent voxels were imprinted in silica glass with different polarization orientations and ellipticities. The maximum retardance of about 5 nm was produced at the ellipticity of 0.6 and no birefringence was detected for a circular polarization [Fig. S2c]. The azimuth of the slow axis of imprinted birefringence was perpendicular to the major axis of the polarization ellipse of the laser writing beam [Fig. S2d].


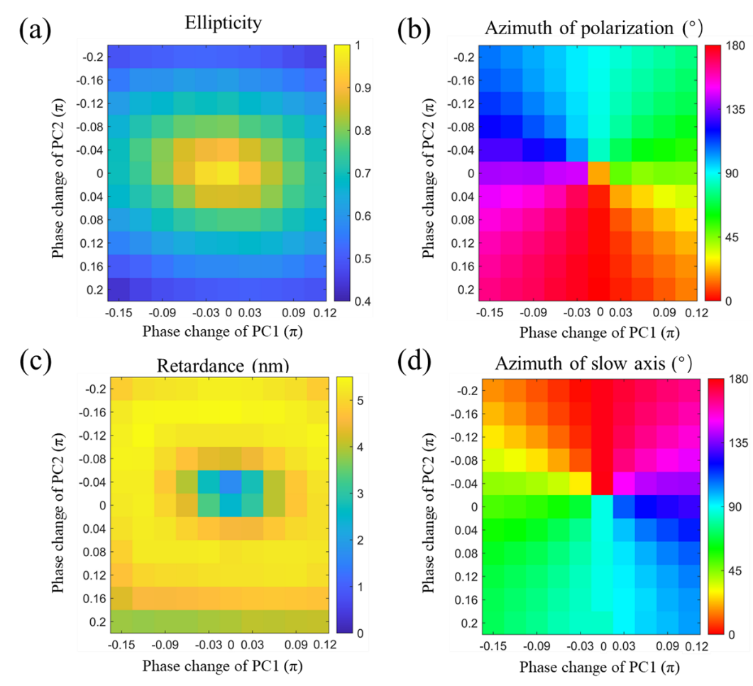


Fig. S2. Elliptical ultrafast laser writing in silica glass by two electro-optical modulators. The measured laser beam ellipticity (a) and the azimuth of polarization (b) at different phase changes of Pockels cells. The measured retardance (c) and the azimuth of slow axis (d) of birefringent voxels written by 20 pulses of 230 nJ with a repetition rate of 1 MHz.

Two retardance levels were produced by polarization ellipticities of 0.6 and 0.8, respectively, as well as 8 azimuths of slow axis, meaning that 4 bits of information (2^4^=2^1^ × 2^3^) can be encoded into one voxel of 5D optical data storage [Fig. S3a]. The readout accuracy of such birefringent voxels is 100% [Fig. S3b], which is ideal for 5D optical data storage. A copy of a digital document was written in 50 layers from bottom to top and the data readout accuracy was nearly 100%.

Compared to data writing with linear polarization, the modulation voltage of Pockels cell required for the elliptical polarization control is reduced 5 times from 5 kV (2π) to about 1 kV (0.4π), resulting in significantly reduced requirements on high voltage amplifiers for high speed operation.


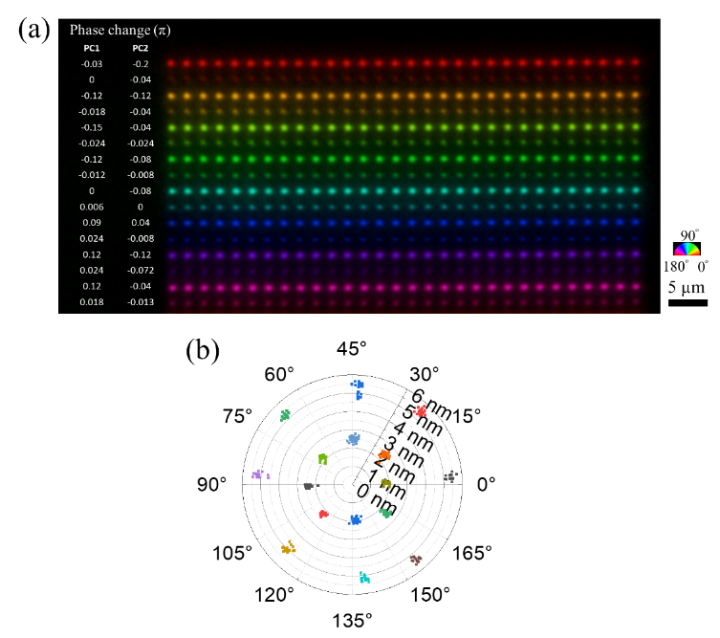


Fig. S3. Elliptical ultrafast laser writing in silica glass by two EOMs. (a) The birefringence image of data voxels for the 2 levels retardance and 8 levels azimuth with different phase change by two EOMs. (b) Polar diagram of measured retardance and azimuth of all voxels in (a). Each voxel was written by 20 pulses of 230 nJ with a repetition rate of 1 MHz and the lateral voxel separation was 2 μm.

1. **Elliptical writing with high repetition rate**

Higher data writing speeds could be achieved by the increase of pulse repetition rate. Type X modification was produced with a fiber amplifier system (Satsuma, Amplitude), operating at 1030 nm with a repetition rate from 1 to 5 MHz and a pulse duration of 270 fs. However, temperature rise and catastrophic damage in the irradiated volume generated by thermal accumulation limits the maximum repetition rate to about 2 MHz for data writing by linearly polarized pulses. The nonlinear absorption of an elliptically polarized pulse is smaller than its linear counterpart, so the thermal degradation at a repetition rate of 5 MHz can be avoided to increase the data write speed [Fig. S4]. It should be noted that the pulse number for each pulse energy varies along vertical direction: 200, 10, 20, 30, 40, 50, 60, 70, 80, 90, 100 and 150.


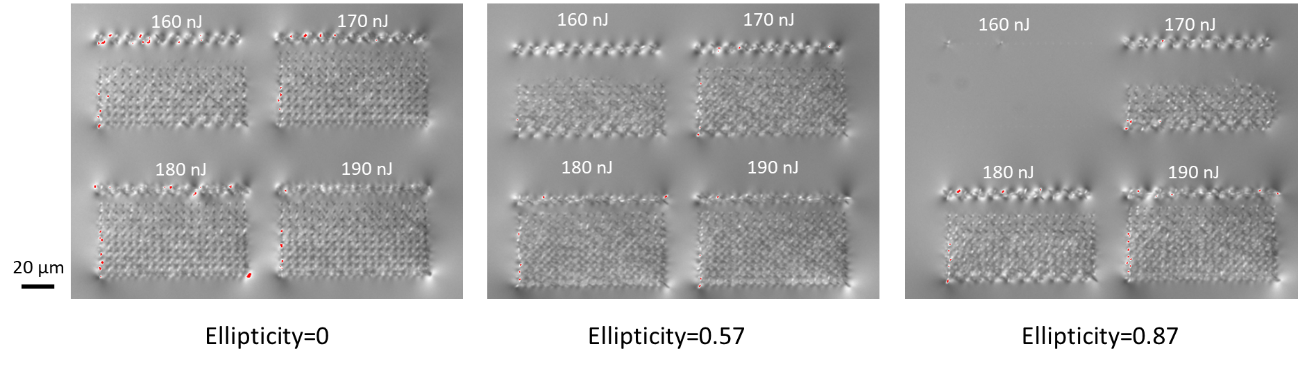


Fig. S4. Optical image of voxels written by different ellipticity of polarization. The voxels imprinted by different pulse numbers and pulse energies with pulse durations of 270 fs, repetition rate of 5 MHz and 0.3 N.A. lens. The lateral voxel separation was 5 μm.

Voxels were written by femtosecond laser pulses with different numbers and energies at repetition rate of 5 MHz. For ellipticity of 0 and 0.57, only thermal damage was observed, and they cannot be used for data storage. However, type X modification was produced with elliptically polarized pulses for pulse energy of 160 nJ and pulse number from 70 to 150, which can be used for 5D optical data storage [Fig. S5].


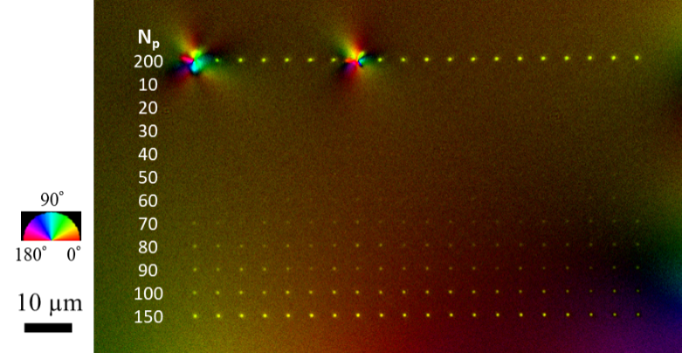


Fig. S5. Birefringence image of ultra-low loss voxels written by different pulse numbers (Np) with ellipticity of 0.87 at repetition rate of 5 MHz and pulse energy of 160 nJ. The lateral voxel separation was 5 μm.

**Reference**

1.Y. Wang, M. Cavillon, N. Ollier, B. Poumellec, and M. Lancry, "An Overview of the Thermal Erasure Mechanisms of Femtosecond Laser‐Induced Nanogratings in Silica Glass," Phys. Status Solidi A **218**, 2100023 (2021).
